# A Shortened Upper Extremity Functional Capacity Evaluation for Patients with Complaints of Hand, Wrist, Forearm, and Elbow: Composition and Assessment of Construct Validity and Test–Retest Reliability

**DOI:** 10.1007/s10926-025-10298-z

**Published:** 2025-05-19

**Authors:** Redmar J. Berduszek, Rienk Dekker, Corry K. van der Sluis, Michiel F. Reneman

**Affiliations:** Department of Rehabilitation Medicine, University Medical Center Groningen, University of Groningen, PO Box 30.001, 9700 RB Groningen, The Netherlands

**Keywords:** Disability, Upper extremity, Functional capacity evaluation, Rehabilitation

## Abstract

**Purpose:**

Upper extremity functional capacity evaluation (UE-FCE) contains tests covering aspects of upper extremity functioning. UE-FCE tests usually consist of multiple repeated trials. Shortened UE-FCEs with less trials per test have been proposed but never tested in patients. The aims of this study were (1) to compose a shortened UE-FCE (fewer trials per test) and (2) to assess construct validity and test–retest reliability when applied in patients with nontraumatic musculoskeletal complaints of the hand, wrist, forearm, and elbow.

**Methods:**

Participants performed a UE-FCE, with original full-length tests, twice (1 to 3 weeks apart). A shortened UE-FCE, with fewer trials per test, was composed based on the agreement (ICC ≥ 0.90) between shortened and original UE-FCE tests. Consequently, construct validity and test–retest reliability of the shortened UE-FCE tests were assessed.

**Results:**

UE-FCEs were performed by 45 participants. The proposed shortened UE-FCE included one-trial tests for hand grip and finger strength (instead of three-trial tests), two-trial tests for fingertip and hand/forearm dexterity (instead of three-trial and four-trial tests, respectively). Overhead lifting and working tests were already one-trial tests and remain unchanged. Construct validity was demonstrated for hand grip strength of the left hand, overhead lifting, and overhead working, but not for hand grip strength of the right hand, finger strength, fingertip dexterity and hand and forearm dexterity. Test–retest reliability was above 0.70 for all tests, except for fingertip dexterity of the dominant hand (0.59).

**Conclusion:**

The shortened UE-FCE with fewer trials per test agreed strongly with the original UE-FCE. Using the shortened UE-FCE could save 18 min. Construct validity differed per UE-FCE test. Test–retest reliability was sufficient for all UE-FCE tests except fingertip dexterity of the dominant hand.

**Supplementary Information:**

The online version contains supplementary material available at 10.1007/s10926-025-10298-z.

## Introduction

Upper extremity musculoskeletal disorders, such as complaints of the arm, neck, and shoulders, are frequently occurring and often have a chronic course [1]. Limitations in daily life and sick leave from work have been reported by over 30% of patients with complaints of the arm, neck, and shoulders [2, 3]. More functional limitations are associated with a poorer prognosis [4]. Functional capacity evaluation (FCE) has been considered relevant in the assessment of functioning of patients with hand disorders [5–7].

FCE is a performance-based measurement to determine what a person can do safely, while considering that person’s body functions and structures, environmental factors, personal factors, and health status [8]. Several FCE protocols, consisting of a different number and type of tests, have been described [9]. Selection of tests depends on the aim of the FCE, considering region or pathology (e.g., lower back, upper extremities, whiplash associated disorder) and/or job requirements (e.g., nurses, household waste collectors) [10–14]. An upper extremity FCE (UE-FCE) consists of tests which cover different aspects of upper extremity functioning, such as joint mobility, muscle power, muscle endurance, and coordination of voluntary movements [7, 15, 16].

Several UE-FCE tests consist of multiple repeated trials, e.g., grip strength and dexterity tests. In healthy subjects, a shorter UE-FCE (with one or two repetitions per test) has been found to be as valid as the original UE-FCE (with three to four repetitions per test) [17]. A shortened UE-FCE is more time-efficient (net time reduction of 33% to 93% per UE-FCE test) and reduces burden on both patient and observer [17]. However, the measurement properties of UE-FCEs with fewer repetitions per test have not yet been assessed in patients.

The aims of this study were (1) to compose a shortened UE-FCE (with fewer repeated trials per test) and (2) to assess construct validity and test–retest reliability of shortened UE-FCE tests when applied in patients with nontraumatic musculoskeletal complaints of the hand, wrist, forearm, and elbow.

## Methods

### Study Design

The design of this observational study was based on the recommendations of the Consensus-based Standards for the selection of health Measurement INstruments (COSMIN) initiative [18, 19]. This study was approved by the Medical Ethical Committee of the University Medical Center Groningen (METc 2015/115) and the protocol has been registered in the Overview of Medical Research in the Netherlands (OMON) register (NL-OMON41867). All participants gave written informed consent.

### Study Sample

Participants were recruited from the outpatient clinic of the department of rehabilitation medicine of a secondary and tertiary care university hospital, between November 2015 and March 2020, using convenience sampling. Inclusion and data collection was terminated prematurely because appointments had to be canceled due to restrictions related to the COVID-19 pandemic. The intended sample size was based on COSMIN recommendations: at least 50 subjects to assess construct validity and reliability [18].

Participants were eligible if they were 18 years or older and had musculoskeletal complaints of their hand, wrist, forearm, and/or elbow, which were not caused by acute trauma or by any systemic disease. These complaints were classified as specific or nonspecific complaints of the arm, neck, and shoulders, according to the classification model for complaints of the arm, neck, and shoulders [20]. From a safety perspective, participants had to meet the criteria of the Physical Activity Readiness Questionnaire (PAR-Q). Because some FCE tasks are physically demanding, the PAR-Q was used to screen for possible risks of physical activity, including cardiovascular and musculoskeletal problems [21]. If PAR-Q question 5 (‘Do you have a bone or joint problem that could be made worse by a change in your physical activity’) was solely answered ‘yes’ because of those complaints of hand, wrist, and/or forearm for which the participant visited the outpatient clinic, we considered the criteria of the PAR-Q were still being met. Exclusion criteria were insufficient understanding of the Dutch language to fill out questionnaires and the presence of concomitant medical conditions causing considerate disability, such as neurological disorders (e.g., stroke, traumatic peripheral nerve damage).

### Procedure

**Fig. 1:**
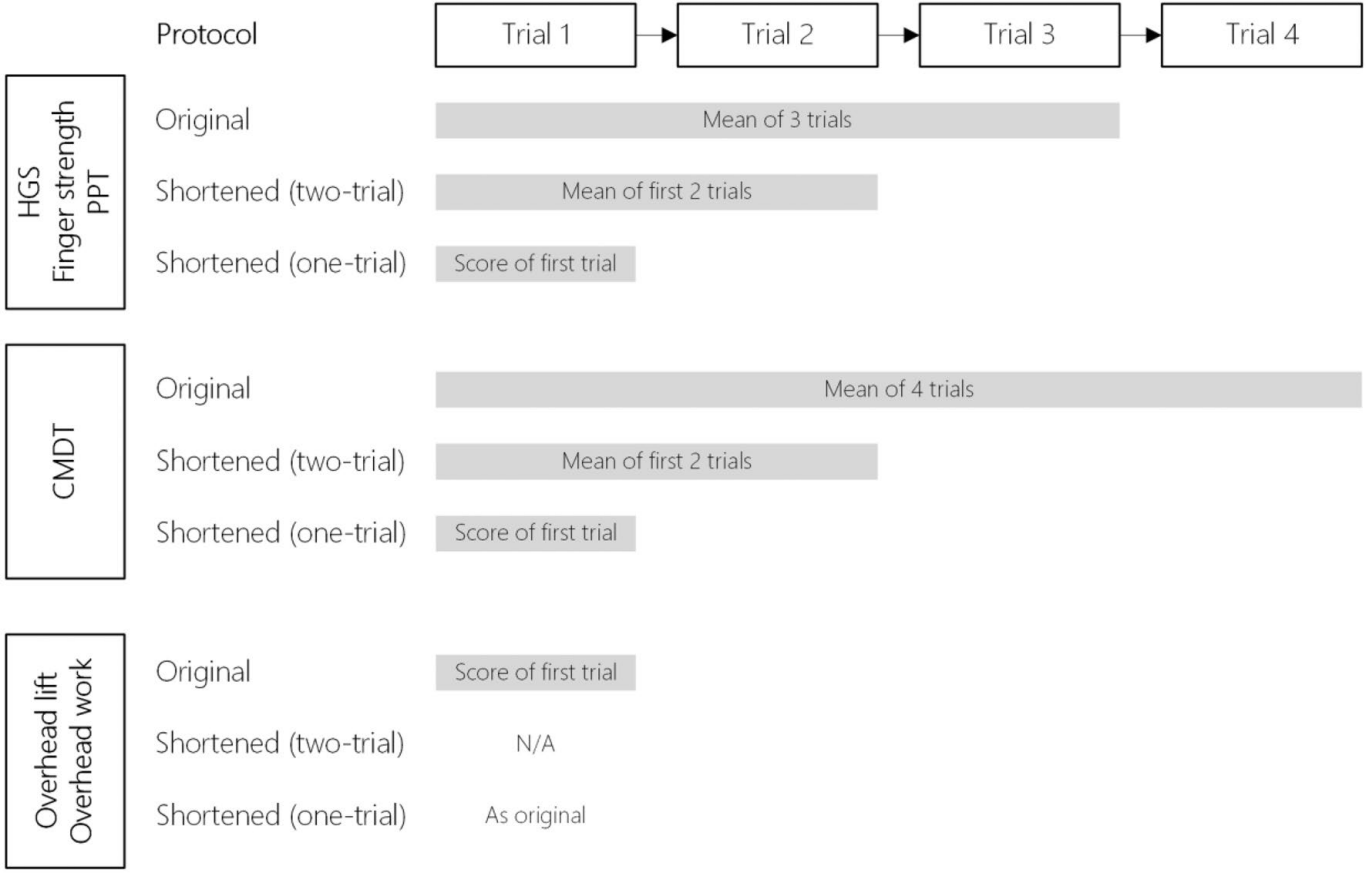
Original and shortened UE-FCE protocols. *HGS* hand grip strength, *PPT* Purdue Pegboard Test, *CMDT* Complete Minnesota Dexterity Test

Measurements were taken during two visits, one to three weeks apart. Questionnaires were filled out prior to each visit. During each visit, a UE-FCE with original full-length tests was administered (Fig. 1). The shortened UE-FCE tests were not administered separately, but their scores were derived from the original UE-FCE test scores. UE-FCEs were administered by one of two FCE-trained certified hand therapists. Both UE-FCEs of an individual participant were observed by the same therapist. During the second visit, therapist and participant were blinded for the results of the first visit. Participants were asked to perform the UE-FCE tests to their maximum ability. Participants were informed that a pain response (increase of already existing complaints or another pain response) could be expected as a normal reaction of the musculoskeletal system to intensive exercise [22].

### Measurements

Demographic information (e.g., level of education, current work status) and patient characteristics (e.g., diagnosis, handedness (dominant hand)) were collected through questionnaires or retrieved from the medical record.

#### Primary Measures: UE-FCE Tests

The UE-FCE consisted of six different tests assessing arm and hand function: hand grip strength, finger strength, fingertip dexterity, hand and forearm dexterity, overhead lifting, and overhead working [15].

##### Hand Grip Strength (Hand Dynamometer)

Hand grip strength (kgf) was measured using a hand dynamometer (Biometrics E-Link). The subject was seated with the shoulder adducted and neutrally rotated, elbow flexed at approximately 90°, forearm and wrist in neutral position. Grip strength of both hands was assessed in five dynamometer handle positions (position 1–5).

##### Finger Strength: Tip, Key and Palmar Pinch Strength (Pinch Grip Dynamometer)

Finger strength (kgf) was measured using a pinch dynamometer (Biometrics E-Link). The subject was seated with the shoulder adducted and neutrally rotated, elbow flexed at approximately 90°, forearm and wrist in neutral position. Finger strength of both hands was assessed in three pinch positions: index fingertip on the thumb tip without facilitation of the middle finger (tip pinch), both index and middle fingertips on the thumb tip (palmar pinch) and thumb pad above the radial side of the index finger (key pinch).

##### Fingertip Dexterity (Purdue Pegboard Test)

Fingertip dexterity was assessed using the Purdue Pegboard Test (PPT). The subject was seated in front of a table with the pegboard on it, placing pins as fast as possible in a 30 s trial. Fingertip dexterity was assessed for both hands. Subjects were given a short practice trial before performing the PPT, to get accustomed to handling the pins and test procedure.

##### Hand and Forearm Dexterity (Complete Minnesota Dexterity Test)

Hand and forearm dexterity was assessed using the Complete Minnesota Dexterity Test (CMDT). The subject was seated in front of a table with the test board on it, displacing 59 blocks in a predetermined way as fast as possible. Hand and forearm dexterity was assessed for both hands. Subjects were given a short practice trial before performing the CMDT, to get accustomed to handling the blocks and test procedure.

##### Overhead Lifting

To assess overhead lifting, the subject had to lift a plastic receptacle from a table to a shelf at crown height and back, repeating this action five times within 90 s while standing. Using weights of 1 kg, 2 kg, and 4 kg, the weight was increased four to five times until the maximum weight was reached. Starting weight, weight increments, and maximum performance were assessed by the test administrator.

##### Overhead Working

To assess overhead working, the subject was standing with hands at crown height, uninterrupted, manipulating nuts and bolts on an aluminum plate with 20 holes for as long as possible. The subject wore cuff weights of 1 kg each around the wrists. The duration for which this position was maintained was measured in seconds.

#### Secondary Measures

The shortened version of the Disabilities of the Arm, Shoulder, and Hand (QuickDASH) is an 11-item questionnaire measuring physical function and symptoms in persons with musculoskeletal disorders of the upper limb. The total score ranges from 0 to 100, a higher score indicating more pain and disability. Adequate measurement properties have been demonstrated in diverse upper extremity disorders, including those of hand and wrist [23–25].

The Patient Rated Wrist/Hand Evaluation (PRWHE) is a 15-item questionnaire measuring pain and disability of the wrist and hand. It consists of two subscales (pain and function) which contribute to the total score equally. The total score ranges from 0 to 100, a higher score indicating more pain and disability. Its measurement properties have been demonstrated to be generally adequate [23, 26].

The Hand Function Sort (HFS) is a 62-item pictorial questionnaire developed to quantify the self-reported physical capacity to perform tasks across a range of physical demands, focusing on upper extremity performance in work tasks and other activities of daily living. An overall rating of perceived capacity score can be calculated with ranges from 0 to 248, a higher score indicating a better capacity. Its measurement properties have been demonstrated to be adequate in patients with complaints of hand and wrist [27].

The Pain Disability Index (PDI) is a 7-item questionnaire measuring the impact of pain on the ability to perform daily activities. The total score ranges from 0 to 70, a higher score indicating greater disability due to pain. Its measurement properties have been demonstrated to be adequate in patients with different types of musculoskeletal pain [28].

The Numeric Pain Rating Scale (NRS Pain) is a valid and reliable single item scale to assess pain intensity during the past week. It is scored on an 11-point Likert scale ranging from 0 (no pain) to 10 (worst pain imaginable) [29].

The RAND 36-item Health Survey (RAND-36) is a valid and reliable questionnaire about physical, mental, and social health and used to measure health-related quality of life. It consists of eight subscales. While the complete RAND-36 was filled out, only the subscales physical functioning, social functioning, vitality and mental health were analyzed in this study. Subscale scores are calculated using an algorithm and range from 0 to 100, a higher score indicating a better health status [30, 31].

### Data Analyses

Statistical analyses were performed using IBM SPSS Statistics 28. Descriptive statistics were used to describe patient characteristics. Statistical significance was set at *p* < 0.05.

#### Part 1: Composing a Shortened UE-FCE

Measurements collected during the first visit were used to evaluate the shortened UE-FCE. The shortened UE-FCE was not tested separately but derived from the data of the original UE-FCE: the one-trial protocol was based on the first trial of the original protocol and the two-trial protocol was based on the mean of the first two trials of the original protocol (Fig. 1) [17]. Agreement between the original and shortened protocols was determined using intraclass correlation coefficients (ICC) for absolute agreement (one-way random effects model). An ICC ≥ 0.90 (indicating excellent agreement) was chosen to reflect sufficient agreement between the original (criterion) and shortened protocols. The final shortened UE-FCE consisted of UE-FCE tests with the least possible number of repetitions per test. Subsequently, measurement properties of this shortened UE-FCE were evaluated.

#### Part 2: Measurement Properties of the Shortened UE-FCE

The scores of the shortened UE-FCE tests were calculated based on the first (number of) attempt(s) of the original full-length UE-FCE tests for both visits (Fig. 1). Measurements collected during the first visit were used to assess construct validity, while measurements from both the first and second visits were used to assess test–retest reliability.

##### Construct Validity

The construct validity of the tests of the shortened UE-FCE was evaluated using predefined hypotheses, based on the concepts being measured.

A total of 11 hypotheses were formulated: nine hypotheses about the relationship with questionnaires assessing hand function, pain and general health and wellbeing, and two hypotheses about differences in performance between men and women and between the symptomatic and asymptomatic side (known-groups validity). The first nine hypotheses were identical for all UE-FCE tests. The latter two hypotheses differed per test group: grip strength tests, dexterity tests and overhead lifting and working.

Strong to moderate correlations were expected with QuickDASH, PRWHE, and HFS, because these questionnaires directly assess self-reported upper extremity function. Moderate correlations were expected with PDI and RAND-36 physical functioning subscale, because these assess broader aspects of daily activities, not primarily influenced by upper extremity complaints. Moderate to weak correlations were expected with RAND-36 social functioning subscale and NRS Pain, because an interaction with hand function was presumed to be present but not explaining all. Weak correlations were expected with RAND-36 subscales for vitality and mental health, because these assess well-being aspects indirectly related to functional capacity.

Regarding sex differences, men were expected to perform better in hand grip tests, overhead working and lifting, due to generally higher muscle mass and strength. No differences between men and women were expected in dexterity tests, as these are less dependent on muscle strength and more on fine motor skills. Regarding differences between the symptomatic and asymptomatic side (in unilaterally affected patients), better performance was expected with the asymptomatic hand compared to the symptomatic hand, based on the assumption that the functional capacity of the affected hand is diminished.

Spearman’s rank correlation coefficients (*r*) were calculated to assess associations with other measurements. Correlation coefficients were interpreted as follows: 0.00–0.25 weak, 0.26–0.50 moderate, 0.51–0.75 strong, above 0.75 very strong [32]. Known-group differences were assessed using Mann–Whitney U tests. Construct validity was sufficient when at least 75% of the results were consistent with the predefined hypotheses [18].

##### Test–Retest Reliability

An ICC for absolute agreement (two-way mixed effects model) was calculated, test–retest reliability was sufficient when ICC ≥ 0.70 [18].

## Results

A total of 72 subjects gave consent to participate and were screened for eligibility. PAR-Q criteria were met by 60 participants who subsequently were included. Two participants were unreachable, two participants were planned for a UE-FCE which could not be carried out because of equipment unavailability, ten participants canceled their appointments stating logistical reasons and the appointments of one participant were canceled because of restrictions related to the COVID-19 pandemic. Of the included participants, 45 participants (75%) performed the original UE-FCE at T1 and 42 participants also performed the original UE-FCE at T2 (see Table 1). Three participants withdrew after T1, mentioning an increase of their complaints following the UE-FCE as the reason.

**Table 1:** Participant characteristics at T1

	Participants who performed UE-FCE at T1 (*n* = 45)	Participants who also performed UE-FCE at T2 (*n* = 42)
Age (years) [mean (SD)]	44.6 (14.9)	44.9 (12.7)
Sex (male) [*n* (%)]	16 (36)	15 (36)
Diagnosis [*n* (%)]		
Specific complaints of the arm, neck, and shoulders	21 (47)	20 (48)
Lateral epicondylitis	6 (29)	5 (25)
De Quervain’s disease	5 (23)	5 (25)
Dupuytren disease	4 (19)	4 (20)
Trigger finger	6 (29)	6 (30)
Nonspecific complaints of the arm, neck, and shoulders	24 (53)	22 (52)
Handedness (dominant hand) [*n* (%)]		
Right-handedness	33 (73)	30 (71)
Left-handedness	9 (20)	9 (21)
Mixed or ambidexterity	3 (7)	3 (7)
Affected side [*n* (%)]		
Unilateral	26 (53)	22 (52)
Bilateral	21 (47)	20 (28)
Dominant hand affected (yes) [*n* (%)]	38 (84)	35 (83)
Employed (yes) [*n* (%)]	34 (76)	31 (78)
QuickDASH [0–100, median (IQR)]	26.1 (17.6)	25.0 (17.1)
PRWHE [0–100, median (IQR)]	34.3 (43.0)	34.0 (41.8)
NRS Pain [0–100, median (IQR)]	4.0 (4.0)	4.0 (4.0)
WAS [0–100, median (IQR)]	7.0 (4.0)	7.0 (4.0)

*UE-FCE *Upper extremity functional capacity evaluation, *QuickDASH* Shortened version of the Disabilities of the Arm, Shoulder, and Hand, *PRWHE *Patient rated wrist/hand evaluation, *NRS* *Pain* numeric pain rating scale, *WAS* work ability score

### Part 1: Composing a Shortened UE-FCE

Results of the agreement between the two shortened protocols (two trials and one trial per test) and the original protocol are shown in Table 2. Hand grip strength was slightly higher in the shortened protocols, except for position 1 of both hands. Dexterity tests were scored better in the original protocol. For all hand grip strength and finger strength tests, the ICC was above 0.90 for both the one-trial and two-trial protocols. For dexterity tests, the ICC was above 0.90 only for the two-trial protocols. Therefore, the proposed shortened UE-FCE is composed of a one-trial test for hand grip strength and finger strength (tip, key and palmar pinch strength) and a two-trial test for fingertip dexterity (PPT) and hand and forearm dexterity (CMDT). Overhead lifting and overhead working were one-trial tests in the original protocol and remained unchanged in the shortened UE-FCE.

**Table 2:** Shortened UE-FCE protocols versus the original UE-FCE protocol

	Original protocol	Shortened (two-trial) versus original protocol		Shortened (one-trial) versus original protocol	
UE-FCE tests	Mean ± SD	Mean difference ± SD	ICC (95% CI**)	Mean difference ± SD	ICC (95% CI**)
Hand grip strength					
HGS pos 1 left	19.6 ± 9.6	− 0.1 ± 1.0	0.99	− 0.5 ± 2.5	0.97 (0.94–0.98)
HGS pos 2 left	27.4 ± 10.4	0.4 ± 0.9*	> 0.99	1.1 ± 2.1*	0.98
HGS pos 3 left	25.9 ± 9.6	0.3 ± 0.7*	> 0.99	0.3 ± 2.0	0.98
HGS pos 4 left	22.3 ± 8.9	0.3 ± 0.7*	> 0.99	0.4 ± 1.8	0.98
HGS pos 5 left	18.9 ± 8.1	0.1 ± 0.6	> 0.99	0.1 ± 1.3	0.99
HGS pos 1 right	20.7 ± 9.6	− 0.1 ± 1.0	> 0.99	− 0.6 ± 1.9*	0.98
HGS pos 2 right	30.1 ± 11.7	0.5 ± 1.0*	> 0.99	0.6 ± 2.0*	0.99
HGS pos 3 right	27.3 ± 10.7	0.3 ± 0.9*	> 0.99	0.9 ± 1.8*	0.98
HGS pos 4 right	23.6 ± 9.6	0.3 ± 0.7*	> 0.99	0.7 ± 1.4*	0.99
HGS pos 5 right	19.7 ± 8.6	0.2 ± 0.7*	> 0.99	0.7 ± 1.5*	0.98
Finger strength					
Tip pinch left	4.1 ± 1.3	− 0.0 ± 0.2	0.99	0.0 ± 0.4	0.97 (0.94–0.98)
Key pinch left	7.1 ± 2.2	− 0.0 ± 0.3	0.99	− 0.1 ± 0.5	0.97 (0.95–0.98)
Palmar pinch left	6.3 ± 2.3	0.0 ± 0.2	> 0.99	− 0.1 ± 0.4	0.98
Tip pinch right	4.3 ± 1.6	0.0 ± 0.2	> 0.99	− 0.0 ± 0.4	0.97 (0.94–0.98)
Key pinch right	7.2 ± 2.3	− 0.0 ± 0.2	> 0.99	− 0.0 ± 0.5	0.98
Palmar pinch right	6.4 ± 2.2	− 0.0 ± 0.3	0.99	− 0.2 ± 0.7	0.95 (0.91–0.97)
Dexterity tests					
PPT dominant	15.2 ± 1.9	− 0.4 ± 0.4*	0.96 (0.92–0.98)	− 1.1 ± 1.2*	0.72 (0.54–0.83)
PPT nondominant	14.2 ± 2.2	− 0.2 ± 0.4*	0.98 (0.97–0.99)	− 0.6 ± 0.6*	0.93 (0.88–0.96)
CMDT dominant	47.9 ± 6.5	1.7 ± 1.2*	0.95 (0.91–0.97)	3.3 ± 2.5*	0.83 (0.71–0.90)
CMDT nondominant	50.9 ± 6.6	1.5 ± 1.6*	0.95 (0.91–0.97)	2.7 ± 2.8*	0.85 (0.74–0.91)

*significant difference (p < 0.05), **95% confidence interval (CI) is only shown when lower limit was 0.95 or lower

*HGS* Hand grip strength, *PPT* Purdue Pegboard Test, *CMDT* Complete Minnesota Dexterity Test

### Part 2: Measurement Properties of the Shortened UE-FCE

#### Construct Validity

For each shortened UE-FCE test, the percentage of results consistent with predefined hypotheses were hand grip strength (left hand 82–100%, right hand 45–64%), finger strength (left hand 36–73%, right hand 27–45%), dexterity tests (PPT 45–73%, CMDT 50–58%), overhead lifting (90%), and overhead working (80%) (Appendix 1). This means that construct validity was demonstrated for hand grip strength of the left hand, overhead lifting, and overhead working, but not for hand grip strength of the right hand, finger strength, fingertip dexterity and hand and forearm dexterity.

#### Test–Retest Reliability

The ICC for test–retest reliability was above 0.70 for all tests, except for fingertip dexterity (PPT) of the dominant hand (0.59) (Table 3).

**Table 3:** Test–retest reliability of the shortened UE-FCE

UE-FCE tests	ICC (95% CI)
Hand grip strength (one-trial)	
HGS position 1 left	0.86 (0.75–0.92)
HGS position 2 left	0.91 (0.84–0.95)
HGS position 3 left	0.94 (0.88–0.96)
HGS position 4 left	0.91 (0.83–0.95)
HGS position 5 left	0.94 (0.89–0.97)
HGS position 1 right	0.85 (0.73–0.91)
HGS position 2 right	0.88 (0.77–0.93)
HGS position 3 right	0.93 (0.87–0.96)
HGS position 4 right	0.95 (0.91–0.97)
HGS position 5 right	0.93 (0.88–0.96)
Finger strength (one-trial)	
Tip pinch left	0.82 (0.69–0.90)
Key pinch left	0.89 (0.81–0.94)
Palmar pinch left	0.79 (0.64–0.88)
Tip pinch right	0.86 (0.76–0.92)
Key pinch right	0.88 (0.77–0.94)
Palmar pinch right	0.78 (0.63–0.88)
Dexterity tests (two-trial)	
PPT dominant	0.59 (0.21–0.79)
PPT nondominant	0.79 (0.51–0.90)
CMDT dominant	0.70 (0.23–0.87)
CMDT nondominant	0.79 (0.46–0.91)
Overhead lifting	0.86 (0.75–0.92)
Overhead working	0.75 (0.58–0.86)

*HGS* Hand grip strength, *PPT* Purdue Pegboard Test, *CMDT* Complete Minnesota Dexterity Test

## Discussion

This study composed a shortened UE-FCE (with fewer repetitions per test) and assessed its measurement properties (construct validity and test–retest reliability) when applied to patients with nontraumatic musculoskeletal complaints of the hand, wrist, forearm, and elbow. A one-trial protocol for hand grip strength and finger strength and a two-trial protocol for dexterity tests (PPT and CMDT) strongly correlated with the original test protocols. Overhead lifting and overhead working were already one-trial tests and remain unchanged in the shortened UE-FCE. Construct validity was confirmed for grip strength of the left hand, overhead lifting, and overhead working. Construct validity of position 2 of the hand dynamometer, which is most commonly used, seemed best for hand grip strength. Less than 75% of hypotheses were confirmed for grip strength of the right hand, finger strength, and dexterity tests. Test–retest reliability of all shortened UE-FCE tests was sufficient, except for the PPT of the dominant hand. However, it should be noted that the confidence intervals of the ICC of some UE-FCE tests, especially the dexterity tests, include 0.70. This indicates that there is a chance that the test–retest reliability of these test may be insufficient. Criteria for interpretation of the ICC values for reliability may differ per study or methodological source (e.g., an ICC of 0.75 instead of 0.70 as used in this study) [33, 34].

Strong correlation between one-trial and original (multi-trial) UE-FCE protocols was already demonstrated in healthy subjects [17]. However, in healthy subjects it was found that a one-trial protocol, instead of a two-trial protocol, was also suitable for dexterity tests. This indicates that dexterity test results vary more over trials in the study population than in healthy subjects. For some UE-FCE tests construct validity could not be demonstrated, mainly due to lower-than-expected correlations with questionnaires assessing hand function (QuickDASH, PRWHE, and HFS). The strongest correlation with these questionnaires was found for overhead lifting and overhead working, possibly because performance during these UE-FCE tests depends on a combination of body functions more than other UE-FCE tests. A remarkable finding was the difference in the percentage of confirmed hypotheses of hand grip strength between the left and right hand, and to a lesser extent of finger strength between the left and the right hand. For these tests, construct validity was much higher for the left hand than for the right hand. Previously, much stronger correlations between either hand grip strength or finger strength and QuickDASH/PRWHE scores were found [35, 36]. There is no obvious explanation for the difference between the left and right hand in these tests, but influence of an interaction between dominant hand and affected hand might be assumed. The fact that in most participants the dominant hand was affected might also explain that no differences were found between the symptomatic and asymptomatic side, where this was expected. Test–retest reliability of the shortened UE-FCE tests for hand grip strength and finger strength, as well as overhead lifting and overhead working, was similar to previous reports about the original protocol in healthy subjects [11]. This indicates that neither fewer trials nor application in the studied target population affected reliability of these tests. However, the test–retest reliability of dexterity tests (both PPT and CMDT) is known to decrease in tests with fewer trials due to learning effects [11, 37, 38]. A learning effect was also observed in dexterity tests administered in this study, despite practicing prior to actually performing these tests, influencing both the validity and test–retest reliability of a shortened test with fewer trials.

### Strengths and Limitations

As far as known, this is the first study assessing a shortened UE-FCE with fewer trials per test in the relevant target population of patients with nontraumatic musculoskeletal complaints of the hand, wrist, forearm, and elbow. The study has been designed and reported in accordance with COSMIN guidelines. Albeit the sample size in this study (45 subjects) was slightly below the COSMIN recommendation (50 subjects) [18], we do not expect relevant differences with a slightly larger sample, given the clear and statistically significant outcomes. However, a larger sample would enable analyzing subgroups based on potentially relevant parameters influencing test results, such as age, sex, handedness (dominant hand), and affected side. About 15% of potential participants could not be included because PAR-Q criteria were not met. It is unlikely that missing these subjects influenced the results, because a relationship between physical fitness and upper limb function was not found in a similar group of patients [39]. Nevertheless, it might hinder generalization to similar patients in clinical practice. No serious adverse events were recorded. A temporary pain response after an FCE, like other forms of physical exercise, is well known and considered a normal reaction [22, 40, 41]. However, three participants ended their participation prematurely because of increased complaints after the first UE-FCE.

### Clinical Implications and Suggestions for Further Research

Using the shortened UE-FCE in clinical practice decreases the time required to administer these tests and lessens the burden on the patient: for the shortened UE-FCE composed in this study this could save an estimated 18 min (total administration time of the shortened UE-FCE tests can be reduced from 29 to 11 min) [17]. Further research should focus on the construct validity of the individual UE-FCE tests, and especially the influence of age, sex, handedness (dominant hand), and affected side. Future research should also assess the structural validity of the (shortened) UE-FCE (e.g., factor analysis and covariance of (groups of) different UE-FCE tests). Depending on the constructs measured, further shortening by means of reducing the number of UE-FCE tests or better guidance toward UE-FCE test selection might be pursued. Also, there is a need to explore the influence of learning effects on the results of dexterity tests and how to deal with that (e.g., expansion and clarification of the number and duration of practice runs before the test trial). Other measurement properties (e.g., responsiveness) should also be evaluated. Eventually, there should be attention to involve patients who were not eligible to participate in this study, specifically those not meeting PAR-Q criteria, as these may represent patients with comorbidities that are relevant for daily clinical practice.

## Conclusion

The shortened UE-FCE with fewer repeated trials per test correlated strongly with the original UE-FCE in patients with nontraumatic musculoskeletal complaints of the hand, wrist, forearm, and elbow. The shortened UE-FCE offers a time-efficient option for the use of UE-FCEs in clinical practice. Construct validity differed per UE-FCE test and was demonstrated for hand grip strength of the left hand, overhead lifting, and overhead working. Test–retest reliability was good for all UE-FCE tests except fingertip dexterity (PPT) of the dominant hand.

## Supplementary Information

Below is the link to the electronic supplementary material.

Supplementary file1 (XLSX 32 KB)

## Data Availability

Datasets generated or analyzed during this study are available from the corresponding author upon reasonable request.

## Abbreviations

FCE: Functional capacity evaluation
UE-FCE: Upper extremity functional capacity evaluation
COSMIN: COnsensus-based Standards for the selection of health Measurement INstruments
PAR-Q: Physical activity readiness questionnaire
PPT: Purdue Pegboard Test
CMDT: Complete Minnesota Dexterity Test
QuickDASH: Shortened version of the disabilities of the arm, shoulder, and hand
PRWHE: Patient rated wrist/hand evaluation
HFS: Hand function sort
PDI: Pain Disability Index
NRS Pain: Numeric pain rating scale
RAND-36: RAND 36-item Health Survey
ICC: Intraclass correlation coefficient
